# Experience of Preimplantation Genetic Diagnosis for Hemophilia at the University Hospital Virgen Del Rocío in Spain: Technical and Clinical Overview

**DOI:** 10.1155/2015/406096

**Published:** 2015-07-16

**Authors:** Raquel M. Fernández, Ana Peciña, Beatriz Sánchez, Maria Dolores Lozano-Arana, Juan Carlos García-Lozano, Rosario Pérez-Garrido, Ramiro Núñez, Salud Borrego, Guillermo Antiñolo

**Affiliations:** ^1^Department of Genetics, Reproduction and Fetal Medicine, Institute of Biomedicine of Seville (IBIS), University Hospital Virgen del Rocío, CSIC, University of Seville, 41013 Seville, Spain; ^2^Centre for Biomedical Network Research on Rare Diseases (CIBERER), 41013 Seville, Spain; ^3^Hemophilia Unit, University Hospital Virgen del Rocío, 41013 Seville, Spain

## Abstract

Hemophilia A and B are the most common hereditary hemorrhagic disorders, with an X-linked mode of inheritance. Reproductive options for the families affected with hemophilia, aiming at the prevention of the birth of children with severe coagulation disorders, include preimplantation genetic diagnosis (PGD). Here we present the results of our PGD Program applied to hemophilia, at the Department of Genetics, Reproduction and Fetal Medicine of the University Hospital Virgen del Rocío in Seville. A total of 34 couples have been included in our program since 2005 (30 for hemophilia A and 4 for hemophilia B). Overall, 60 cycles were performed, providing a total of 508 embryos. The overall percentage of transfers per cycle was 81.7% and the live birth rate per cycle ranged from 10.3 to 24.1% depending on the methodological approach applied. Although PGD for hemophilia can be focused on gender selection of female embryos, our results demonstrate that methodological approaches that allow the diagnosis of the hemophilia status of every embryo have notorious advantages. Our PGD Program resulted in the birth of 12 healthy babies for 10 out of the 34 couples (29.4%), constituting a relevant achievement for the Spanish Public Health System within the field of haematological disorders.

## 1. Introduction

Hemophilia A and B (OMIM #306700 and #306900), inherited as X-linked recessive traits, are the most common hereditary hemorrhagic disorders caused by a deficiency or dysfunction of blood coagulation factor VIII (FVIII) and factor IX (FIX). These diseases have an incidence of 1 in 5000 and 1 in 25000 male births, respectively, and no ethnic or geographical predisposition has been described. Both types of hemophilia, clinically indistinguishable, are characterized by deficiency in FVIII or FIX clotting activity that results in prolonged oozing after injuries or surgery and delayed or recurrent bleeding prior to complete wound healing. The major cause of disability from bleeding is chronic joint disease [1]. Currently, available treatment with clotting factor concentrates is normalizing life expectancy and reducing chronic joint disease for children and adults with hemophilia. For instance, prior to the availability of such treatment, the median life expectancy for individuals with severe hemophilia A was 11 years, while new life expectancy for those severely affected individuals receiving adequate treatment is 63 years [2].

The age of diagnosis and frequency of bleeding episodes are related to the level of FVIII or FIX clotting activity, which is in close association with the mutation at either the* F8 *gene (encoding FVIII) or* F9 *gene (encoding FIX) responsible for the disease in each case. Therefore, the clinical presentation of both types of hemophilia in males can vary from severe to moderate or mild, while the majority of carrier females (around 90%) are asymptomatic.

There is a 50% chance that a carrier mother transmits the defective X-linked gene to her children, and in such circumstances the female offspring will be also carrier of the disease, while the male offspring will be clinically affected. On the other hand, all female offspring born to a hemophilic father are obligatory carriers, while the male offspring will be free of inheriting the mutant X-linked gene.

Both* F8* and* F9* genes map to the long arm of X-chromosome at Xq28 and Xq27, separated by 35 cM [3–5]. An* F8* intron 22-A inversion is identified in nearly half of families with severe hemophilia A [6], an intron 1 inversion is identified in 2%–5% of the patients, and the remaining cases are due to other point pathogenic variants that may include small intragenic deletions/insertions and missense, nonsense, and splice site variants along the* F8* gene. In contrast, in moderate to mild hemophilia A, such inversions are not detected and 76–99% of the cases have been reported to be caused by* F8* point mutations [6–9] (GeneReviews for hemophilia A; http://www.ncbi.nlm.nih.gov/books/NBK1404/).

Regarding hemophilia B,* F9* large gene deletions, nonsense mutations, and frameshift mutations cause severe disease, while missense mutations can cause severe, moderate, or mild disease depending on their location and the specific substitutions involved [10, 11] (GeneReviews for hemophilia B; http://www.ncbi.nlm.nih.gov/books/NBK1495/).

In this scenario reproductive options for the families affected with hemophilia include different options. They may decide not to have children, foster, or adopt. Another option is to attempt natural conception and either to accept the risks of giving birth to an affected child or to have prenatal diagnosis followed by possible termination of an affected pregnancy. Since the decision to whether or not terminate pregnancy is frequently confronted to the religious, ethical, and cultural points of view of families, couples may choose to have a Preimplantation Genetic Diagnosis (PGD) cycle. This is an advanced assisted conception technique that gives couples the chance of giving birth to an unaffected child of a specific disease, although the requirement of in vitro fertilization (IVF) procedures involves risks of ovarian hyperstimulation syndrome and multiple pregnancy and may increase the risk of birth defects not related to the disease tested [12].

Here we present the results of our program of PGD of hemophilia A and B. All the procedures were performed at the University Hospital Virgen del Rocío in Sevilla, Spain (HUVR).

## 2. Materials and Methods

### 2.1. Inclusion of Couples in Our PGD Program for Hemophilia A and B

Since 2005, a total of 36 couples requested their inclusion in our PGD Program for the selection of embryos free of hemophilia. Of them, 2 couples were excluded of our program for different reasons and 34 couples were finally attended in our department (30 couples for hemophilia A and 4 couples for hemophilia B). During the first consultation, the couples should provide a clear and accurate genetic test report, confirming the carrier status for hemophilia for the female and specifying the* F8* or* F9* mutation responsible for the disease in the context of that family. Exceptionally, there is no requirement of a previous genetic study for the obligate carriers of hemophilia (daughters of affected males). Extensive genetic counselling and information about the PGD procedures, success rate, and possibility of misdiagnosis inherent to techniques are then given by our multidisciplinary team of geneticists, embryologists, and gynaecologists to the couple. Informed consent concerning PGD and related procedures as well as the fate of the nontransferred embryos must be signed by the couples. Then, a basic test is prescribed to evaluate the reproductive status of the couples, which includes hormone analysis and transvaginal ultrasound in the female, seminogram in the male, and serology for hepatitis B and C, HIV, and syphilis in both of them.

Since 2009, PGD of hemophilia is carried out in our center by indirect molecular analysis using short tandem repeats (STRs) located in the intragenic or neighbouring regions to the* F8* gene (in case of hemophilia A), or along the X and Y chromosomes in general (in case of hemophilia B). Once the results of the basic test meet the minimal quality requirements for the assisted reproduction techniques, DNA samples are extracted from peripheral blood of both members of the couple and other family members (usually an affected male) to proceed with the selection of informative genetic markers in the context of each family. Finally, when such selection has been achieved and the “disease haplotype” has been clearly identified, warranting the reliable diagnosis of future embryos, the couple starts with the assisted reproduction protocol. Assisted reproductive techniques followed in our center have been previously described [13–15].

### 2.2. Fluorescent In Situ Hybridization (FISH) Analysis for Selection of Female Embryos

From 2005 to 2009, in our center, PGD for hemophilia was based on the selection of female embryos, using FISH analysis. Embryo biopsy procedures are performed as previously reported [13–15]. Single blastomeres were fixed using Tween 20/HCl and Carnoy's solution as per the method described by Dozortsev and McGinnis [16]. Fixed slides were treated using the protocol recommended by Vysis Inc. The FISH analysis for chromosomes X, Y, 13, 18, and 21 was carried out using the Vysis MultiVysion PGT Multi-Color Probe Kit (Abbot Molecular) and following the recommendations of the manufacturers. Slides were hybridized in a HYBrite (Vysis Inc.) using the optimised FISH conditions. Posthybridization washes were performed and Antifade II counterstain (Vysis Inc.) was added. Nuclei were analysed independently by two PGD scientists using an Olympus BX51 fluorescent microscope (Olympus Optical Co. Ltd., Tokyo, Japan) and CytoVision v4.0 with the GSL-120 software.

### 2.3. Selection of Markers for Indirect Molecular Diagnosis of Hemophilia A and Multiplex PCR Protocol

A panel of six polymorphic STRs located within the* F8* gene or closely surrounding it was selected to perform indirect molecular analysis of hemophilia A.

Five of the markers (*DXS9901*,* DXS8087*,* DXS1073*,* STR13*, and* DXS1108*) had been used for PGD of HA and other diseases mapped in the Xq28 chromosomal region [17]. Subsequently, a selection of 4 markers (*DXS1073*,* STR13*,* STR22*, and* DXS1108*) were described to offer a high heterozygosity rate (>90%) that improves tracing of* F8* gene inheritance [18], and therefore we prioritized its use whenever possible. Specifically,* STR13* consists of a TG repeat located in intron 13, and* STR22* consists of a GT repeat in intron 22.* DXS1073* (TG repeat) and* DXS1108 *(CA repeat) are extragenic STRs closely linked to the* F8* gene and are located ~235 kb centromeric and ~610 kb telomeric to* F8*, respectively. Sequences of the primers for the amplification of those markers were previously reported [18]. Just in case that any of the markers was not informative in the context of a family, we used the remaining two markers,* DXS9901* and* DXS8087*. Primers for the amplification of those 2 markers were designed using the Primer3 software (http://primer3.sourceforge.net/) so that they could be amplified with the same annealing temperature and conditions than the other 4 STRs.

A one-step multiplex single-cell fluorescent PCR is used for the simultaneous amplification of several combinations of the 6 markers, using the QIAGEN Multiplex PCR kit (QIAGEN, GmbH; Hilden, Germany) and an adaptation from a protocol previously described [13]. Optimal cell lysis protocol and PCR conditions, further described, were set up on single cells biopsied from supernumerary IVF embryos not suitable for transfer or cryopreservation. The optimized reaction mix contains 0,2 *μ*M of each primer, 5x Sol Q, and 2x QIAGEN Multiplex PCR Master Mix, for a final volume of 25 *μ*L. The PCR program is as follows: 15 minutes at 94°C, 10 cycles of 30 seconds at 96°C, 30 seconds at 55°C, 1 minute at 72°C, followed by 35 cycles of 30 seconds at 94°C, 30 seconds at 55°C, 1 minute at 72°C, and a final extension of 15 minutes at 60°C. PCR products are analyzed on an ABI3730 automated sequencer (Applied Biosystems, Foster City, CA).

### 2.4. Selection of Markers for Embryo Sexing (Applied to PGD of Hemophilia B) 

Since the number of couples requesting a PGD cycle for hemophilia B is very low, for cost-effective reasons, we were limited to perform an embryo sexing procedure in order to select females. Cells were subjected to multiplex PCR amplification using oligonucleotides designed for amplification of different fragments of both chromosomes X and Y: SRY, ZFY, AMELX, and AMELY. Different combinations of intragenic markers of the* DMD* gene, located at Xp21.2 (*STR44*,* STR45*,* STR49*, or* STR50*) are also added to the mix. The specific combination of markers selected for each PGD cycle depends on their heterozygosity in the context of each couple. Primers sequences are available on request.

The optimized reaction mix contains 0,2 *μ*M of each primer, 5x Sol Q, and 2x QIAGEN Multiplex PCR Master Mix, for a final volume of 15 *μ*L. The PCR program is as follows: 15 minutes at 95°C, followed by 40 cycles of 30 seconds at 95°C, 1 minute at 58°C and 1 minute at 72°C, and a final extension of 15 minutes at 60°C.

### 2.5. Haplotyping of the Embryos

After 30 min. at −80°C, cells are lysed by incubation in SDS (17*μ*M)/proteinase K (125 *μ*g/mL) lysis buffer for 90 min. at 37°C and 15 min. at 65°C.

The corresponding genetic analysis of the embryos is subsequently performed using the previously selected combination of markers and the described one-step multiplex fluorescent PCR protocol at the single-cell level.

## 3. Results 

### 3.1. Overall Clinical Results

At the start of the PGD Program, two cells were taken from each embryo in order to verify the results, but once we experienced that a conclusive and reliable diagnosis for the embryos could be obtained on the basis of one cell, we limited the cells taken from each embryo to one.

The overall clinical results for the PGD cycles for hemophilia A and B are summarized in Table 1. Following the policies of our institution, a maximum of 3 cycles were performed per couple. In general, response to hormonal stimulation was quite high in almost all the cases in comparison to the couples with fertility problems attended in our center, as it should be expected. In general, the number of mature oocytes submitted to ICSI per cycle, although highly variable depending on the couple, was quite satisfactory (13.0 ± 6.2* versus *6.7 ± 3.2 for couples with reproductive problems). Moreover, the overall number of oocytes fertilized per cycle was 8.6 ± 4.7, in comparison with the 3.7 ± 2.3 obtained for infertile couples.

In the case of hemophilia A, a total of 53 cycles were performed for 30 couples. Only 2 out of the 53 cycles resulted in no embryos; both of them were performed for the same couple. Molecular analyses of the embryos resulting from the remaining 51 cycles were performed by FISH in the case of 11 of the couples (20 cycles), by fluorescent multiplex PCR in 17 couples (28 cycles), and by a combination of both techniques in 1 couple (2 cycles by FISH and a third cycle by PCR). Independently of the molecular method applied for the analysis of embryos, the fertilization rate, considered as the correctly fertilized oocytes out of the total number of mature injected oocytes, was 65.6% (451 out of 688 oocytes). A total of 337 out of the 451 embryos were analyzed (74.7%), with a very variable number of embryos analysed per cycle, ranging from 0 to 18, mainly depending on the characteristics of the couple treated. 284 out of the 337 analysed embryos (84.3%) were reliably diagnosed. Finally, 72 embryos were transferred in 43 out of the 58 cycles, which corresponds to a transfer rate of 74.1% (Table 1). Biochemical pregnancy (considered when *β*-hCG values ≥5 UI/L, 9 days after transfer) was achieved in 18 of those 43 cycles (41.9%), for 14 of the 30 couples (46.7%). Pregnancy was subsequently confirmed by echography in 10 of the 30 couples (33.3%), leading to clinical pregnancy rate of 17.2% per initiated cycle and of 23.3% per transfer. One miscarriage occurred during the first trimester, leading to the birth at term of 10 unaffected children for 9 couples, which corresponds to a live birth rate of 15.5% per initiated cycle and of 20.9% per transfer.

Regarding hemophilia B, a total of 7 cycles for 4 couples were performed. Irrespective of the molecular technique used for embryo sexing, the fertilization rate was 71.3% (57 out of 80 mature injected oocytes). The overall number of embryos analysed per cycle (46 out of the 57 embryos, 80.7%) ranged from 2 to 11. Accurate molecular results were obtained for 39 out of the 46 analysed embryos (84.8%). Finally, 11 female embryos were transferred in 6 out of the 7 cycles, which corresponds to a transfer rate of 85.7% (Table 1). In 1 of the 4 couples (25%), efforts resulted in a successful pregnancy after just 1 PGD cycle, with the birth at term of two girls. Therefore, both the clinical pregnancy and live birth rates were 14.3% per initiated cycle and 16.7% per transfer.

Overall, taking into account both hemophilia A and B, 34 couples were treated in 60 cycles and 10 couples (9 for hemophilia A and 1 for hemophilia B, 29.4%) had unaffected babies (live birth rate of 20.40% per transfer and of 16.7% per initiated cycle).

### 3.2. Clinical Results Obtained When Using FISH for Embryo Sexing 

During the period of time in which we applied FISH for embryo sexing to select female embryos, we performed a total 23 cycles for 14 couples (6 couples with 1 cycle, 7 couples with 2 cycles, and 1 couple with 3 cycles). The reason of inclusion was hemophilia A for 10 couples and hemophilia B for the remaining 2 couples. The overall percentage of embryos whose gender could not be determined was 22.8% (31 out of 136 embryos). Difficulties inherent to the FISH technical procedures or the impossibility to find the fixed hybridized single blastomeres in the slides were the main causes of this percentage. Among the 105 informative embryos, a total of 21 (20%) were reported to have any kind of chromosomal anomaly detected by FISH or to present inconclusive results regarding their status for chromosomes 13, 18, and 21, and therefore they were discarded for further transfer. Finally, 53 out of the 84 remaining embryos were males (63%) and 31 were females (37%). Embryo transfer was achieved in 18 of these 23 cycles (78.3%). Specifically, 2 embryos were transferred in 8 cycles resulting in a biochemical pregnancy in one of them (12.5%), and just 1 embryo was transferred in the remaining 10 cycles, resulting in a biochemical pregnancy in 5 of them (50%) that progressed to clinical pregnancy in 3 cases (30%). Overall, the transfer rate when using FISH for females selection was 78.3%, the clinical pregnancy rate per transfer was 16.6%, and the clinical pregnancy rate per cycle was 13.0%. The 3 clinical pregnancies (all of them of female carriers of hemophilia A, 21.4% of the couples) resulted in the successful birth of 3 girls.

### 3.3. Clinical Results Obtained When Using Fluorescent Multiplex PCR for Embryo Sexing

Since 2009, we began to use the genotyping of STRs by fluorescent PCR for embryo sexing applied to hemophilia B. Four cycles for 2 couples were performed (1 couple with just 1 cycle and another with 3 cycles). Using this approach, the percentage of embryos with undetermined gender decreased to 17.4% (4 out of 23 embryos). Among the 19 informative embryos, 10 were males (52.6%) and 9 were females (47.4%). Embryo transfer was attained in 3 of these 4 cycles, reaching a transfer rate of 75%. Two embryos were transferred in each of those 3 cycles resulting in both biochemical and clinical pregnancy and subsequent birth of 2 girls in one of them, which leads to a clinical pregnancy rate per transfer of 33.3% and a clinical pregnancy rate per cycle of 25.0%.

### 3.4. Clinical Results Obtained When Using Fluorescent Multiplex PCR for Specific Diagnosis of Hemophilia A of the Embryos

**Figure 1:**
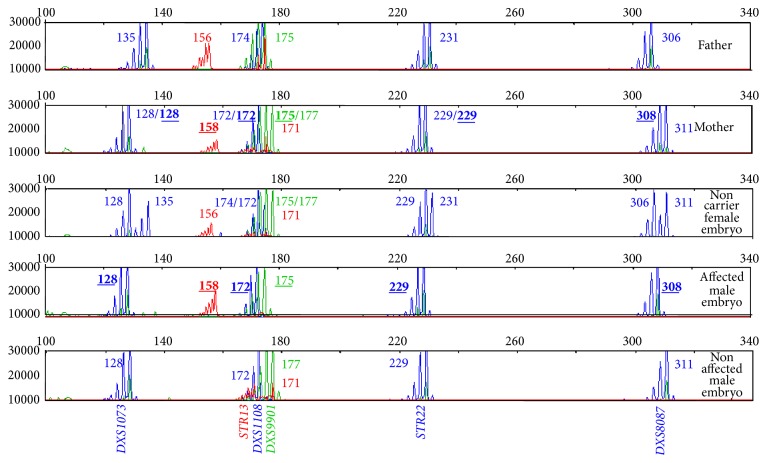
Electropherograms showing the profile of the STR markers throughout the* F8 *gene and its surrounding regions. Each lane shows the peaks obtained for each marker after the application of the specific multiplex fluorescent PCR method for hemophilia in a couple (lanes 1 and 2) and in the blastomeres biopsied from some of the embryos of their PGD cycle. Each peak corresponds to the PCR product amplification of a marker, whose size depends on the specific number of repeats (the more the repeats the larger the PCR product). The sizes for each marker in each lane are also indicated with numbers. The sizes of the specific combination of alleles linked to the disease, which was identified by a previous informativity testing in the context of the family, are shown in bold and underlined characters (lanes 2 and 4).

Regarding the use of the specific multiplex fluorescent PCR method applied to the molecular diagnosis of hemophilia A (Figure 1), it was applied to a total of 205 embryos resulting from 31 cycles on 19 couples (9 couples with 1 cycle, 6 couples with 2 cycles, 3 couples with 3 cycles, and another couple with 2 previous cycles performed with FISH).

The percentage of undiagnosed embryos was 12.2% (25 out of 205 embryos). While we did not detect any allele dropout for single markers selected in each case, undiagnosis was due to no amplification of the whole set of markers for some blastomeres. Contaminations with other sources of DNA were not detected either. Taking into account exclusively the embryos with a conclusive diagnosis for HA by indirect molecular analysis, the number of transferable embryos was 122 (67.8%) (38 unaffected males, 45 noncarrier females, and 39 carrier females). Embryo transfer was attained in 26 of the 31 cycles, reaching a transfer rate of 83.9%. Two embryos were transferred in 22 cycles resulting in biochemical pregnancy in 10 cycles (45.5%) that progressed to clinical pregnancy and successful birth of children in 7 cases (31.8%). Specifically, just one of the 7 clinical pregnancies resulted in the delivery of 2 healthy boys, and the remaining 6 concluded in the birth of singletons (2 unaffected boys and 4 girls). Regarding the 4 cycles in which just 1 embryo was transferred, biochemical but no clinical pregnancy was achieved in 2 of them (50%). Overall, when using this specific method for PGD on hemophilia A, the clinical pregnancy and live birth rates per transfer were 26.9%, and the clinical pregnancy and live birth rates per cycle were 22.6%.

Remaining unaffected embryos resulting from all the cycles that did not achieve enough quality to be transferred or cryopreserved, as well as the affected embryos, were retested for the corresponding markers in each case, and the initial results were confirmed in all of them. The unaffected embryos suitable to be cryopreserved were vitrified using the Vit Kit Freeze kit (Irvine Scientific) and the protocol provided by the manufacturers.

## 4. Discussion

PGD offers an alternative for the couples who are unwilling to accept prenatal diagnosis leading to possible termination of pregnancy. As a general rule in Spain, preimplantation genetic testing techniques are not usually paid by public healthcare. However, in 2005, the Andalusia Regional Government authorized PGD for a closed list of specific monogenic diseases (156/2005 decree, of June 28th), including X-linked disorders such as hemophilia A and B. PGD became therefore accessible through the public healthcare system and specifically through the HUVR in Seville, one of the leading centers for genetic-based research in Spain. HUVR was the first public center offering PGD in Spain, although currently another three public centers offer it. Together with the undeniable advantages for the families of having unaffected offspring of hemophilia, the PGD Program is also cost-effective: while the approximate cost for a PGD cycle in our institution is around €3.500 (unpublished data), the mean cost of treatment per bleed has been estimated at €8.473–€8.627 and €15.579–€15.677 in children and adults, respectively [19].

Currently, many clinics offer PGD for hemophilia, using a strategy applied to a range of X-linked recessive disorders, that is, gender selection by FISH or PCR and transfer of only female embryos. In fact, our institution still performs embryo sexing to select female embryos for couples affected by hemophilia B, since to date the number of couples that requested their inclusion in our PGD Program for the selection of embryos free of HB has been very low (4 couples). The development of a PCR protocol to amplify several markers along the sex chromosomes in a single reaction has provided a faster, safer, and more economical way to determine the sex of embryos for PGD of hemophilia B than FISH. Notorious differences can be observed among the results after the application of the two techniques (PCR* versus* FISH) for embryo sexing: 82.6%* versus* 80.0% of informative embryos, 12%* versus* 25% of clinical pregnancies per cycle, and 8%* versus* 25% of live birth rates per cycle.

A completely different scenario is observed for HA, for which requests of inclusion in our PGD Program comprise the 16.3% of our total requests. In such situation, an analysis that allows the diagnosis of the HA status of every embryo (i.e., affected male, carrier female, or normal/not affected) is more desirable because the number of embryos available for transfer from each cycle is increased (75%, as compared with 50% for females only). In addition, couples prefer to know that they have the possibility of conceiving a healthy child of either gender. Specific DNA diagnosis has therefore two important advantages: firstly, healthy male embryos are not discarded and secondly, female carriers can be identified. In fact, the risk of skewed X inactivation and the possibility of hemophilia occurring in a female carrier, although infrequent, still exist, and our strategy would make it possible to prioritize the transfer of unaffected males and noncarrier females* versus* carrier females, whenever the number and quality of transferable embryos are enough. In our own experience, our data demonstrate a clear improvement in our clinical results after the introduction of our specific PCR method of PGD of HA. First, the % of cycles with transfer has increased around 10% (89.7% of transfers with specific PGD-HA method* versus* 79.3% with embryo sexing). Second, the % of clinical pregnancies per cycle is also consequently almost double higher (24.1% with the PGD-HA method* versus *13.8% with embryo sexing). Finally, and most important, the live birth rate per cycle increased from 10.3% obtained with embryo sexing to 24.1% achieved with the PGD-HA PCR-based method.

These notorious differences in our results among both approaches (PGD-HA specific method* versus* embryo sexing) are related not only to the fact that the theoretical % of transferable embryos is 75%* versus* 50%, but also to the robustness, efficiency, and accuracy of our multiplex PCR-based method* per se*. This is clearly reflected by the percentage of informative embryos obtained with each method: 80.0% with embryo sexing by FISH* versus* 87.8% with the PGD-HA method. Since tracking of the “disease chromosome” can be performed independently of the precise mutation causing the disease, indirect testing can be used for diseases where the disease is caused by a great number of different mutations in a large gene, as in the case of HA. Predictive accuracy for indirect testing, avoiding misdetection of recombination events, is complete when intragenic or closely flanking markers are used, as it occurs with our method, which permits the inclusion of highly heterozygous intragenic and/or flanking markers. Among the advantages of our method, we could emphasize that it is highly versatile making it possible to simultaneously amplify several different combinations of STR markers (depending on the couple) and that it just involves a round of PCR with the subsequent reduction of the contamination possibility and of the work-up time to finally obtain a proper typing and diagnosis (around 3 hours after the embryos biopsy). Moreover, no ADO events have been detected in comparison with other methods reported elsewhere. Nevertheless, the possibility that the markers are uninformative for segregation analysis in the future couples requesting PGD for hemophilia A exists. Therefore, despite the simplicity, cost-effectiveness, and efficiency of the haplotype analysis, we keep in mind implementing direct methods for mutation detection in our center to apply them when necessary. In fact we have already optimized the technique of Whole Genome Amplification (WGA) based on Multiple Displacement Amplification (MDA) on blastomeres, which let us obtain enough DNA quantity to perform a wide spectrum of independent genetic analyses such as sequencing [15].

On the other hand, our clinical results are in general quite similar to those obtained by other centers. In 2012, the ESHRE PGD Consortium reported the results of 10 years of data collection from several international PGD centres [20]. In general, a total number of 1167 cycles for sexing for X-linked disease were reported during their first 10 years of data collection leading to a clinical pregnancy rate of 26% per transfer (27% in the case of hemophilia), quite similar to the overall 22.5% achieved in our Institution (from 15% achieved by embryo selection with FISH to 26.9% obtained by the specific PGD-HA PCR-based method).

## 5. Conclusions

In summary, the balance of our PGD Program applied to hemophilia since 2005, leading to the birth of unaffected babies for 10 of the 34 couples treated (29.4%), has quite satisfactory outcomes, constituting a relevant advance within the field of hematological disorders in the Spanish Public Health System.

## Figures and Tables

**Table 1:** Clinical data for PGD of hemophilia at HUVR.

	No embryos obtained	Embryo sexing by FISH	Embryo sexing by PCR	Embryo sexing in general	Specific PCR method for HA	Total
Number of couples treated	1	14^*∗*^	2^*∗*^	16^*∗*^	18	34^*∗*^
Maternal age	40	31.0 ± 4.9	30.5 ± 3.5		31.8 ± 2.9	31.6 ± 4.1
Number of cycles performed	2	25	4	29	29	60
Number of cycles performed per couple	2	1.9 ± 0.8	2.0 ± 1.4	1.9 ± 0.8	1.7 ± 0.8	1.8 ± 0.8
Number of mature oocytes submitted to ICSI	7	317 (280 HA + 37 HB)	43 (HB)	360 (280 HA + 80 HB)	408	775
Number of mature oocytes submitted to ICSI per cycle	3.5 ± 4.9	12.7 ± 4.7	10.8 ± 5.1	12.4 ± 4.7	13.6 ± 7.3	13.0 ± 6.2
Number of oocytes fertilized	0	217 (187 HA + 30 HB)	27 (HB)	244 (187 HA + 57 HB)	264	508
% of oocytes fertilized	—	68.5%	62.8%	67.8%	64.7%	65.6%
Number of oocytes fertilized per cycle	—	8.7 ± 3.5	6.8 ± 3.4	8.4 ± 3.5	8.8 ± 5.6	8.6 ± 4.7
Number of embryos analyzed	—	155 (132 HA + 23 HB)	23 (HB)	178 (132 HA + 46 HB)	205	383
% of embryos analyzed	—	71.4%	85.2%	73.0%	77.7%	75.4%
Number of embryos analyzed per cycle	—	6.2 ± 2.3	5.8 ± 2.5	6.1 ± 2.3	6.8 ± 4.5	6.5 ± 3.6
Number of informative embryos	—	124 (104 HA + 20 HB)	19	143 (104 HA + 39 HB)	180	323
% of informative embryos	—	**80.0%**	**82.6%**	**80.3%**	**87.8%**	84.3%
Number of transfers	—	20 (17 HA + 3 HB)	3 (HB)	23 (17 HA + 6 HB)	26	49
% of transfers	—	**80.0%**	**75.0%**	**79.3%**	**89.7%**	81.7%
Number of embryos transferred	—	29 (24 HA + 5 HB)	6	35 (24 HA + 11 HB)	48	83
Number of biochemical pregnancies	—	6 (6 HA)	1 (HB)	7 (6 HA + 1 HB)	12	19
Number of clinical pregnancies	—	3 (3 HA)	1 (HB)	4 (3 HA + 1 HB)	7	11
% of clinical pregnancies per cycle	—	**12.0%**	**25.0%**	**13.8%**	**24.1%**	18.3%
% of clinical pregnancies per transfer	—	15.0%	33.3%	17.4%	26.9%	22.5%
Implantation rate	—	10.3%	33.3%	14.3%	16.7%	15.7%
Number of pregnancies went to term	—	2	1	3	7	10
Number of babies born	—	2	2	4	8	12
Live birth rate per cycle	—	**8.0%**	**25.0%**	**10.3%**	**24.1%**	16.7%

^*∗*^One of the couples was submitted to two PGD cycles using FISH for embryo sexing and to a third cycle using the specific multiplex PCR method for hemophilia A.
